# DNA Methylation Regulatory Axis miR‐29b‐3p/DNMT3B Regulates Liver Regeneration Process by Altering LATS1

**DOI:** 10.1111/jcmm.70405

**Published:** 2025-02-12

**Authors:** Yinwen Zhou, Hao Wu, Qiu Wang, Bo Ma, Jiulong Sun, Guoliang Wang

**Affiliations:** ^1^ Department of Hepatobiliary Surgery and Organ Transplantation Guizhou Provincial People's Hospital Guiyang Guizhou China; ^2^ Department of Hepatobiliary Surgery Zunyi Medical University Zunyi Guizhou China; ^3^ Division of Breast Surgery, Department of General Surgery, Breast Center, West China Hospital Sichuan University Chengdu Sichuan China

**Keywords:** DNA methylation, LATS1, liver regeneration, miR‐29b‐3p/DNMT3B axis

## Abstract

DNA methylation is a crucial epigenetic alteration involved in diverse biological processes and diseases. Hippo signalling pathway is a key signalling regulatory network in the growth and development of tissues and organs. Nevertheless, the precise role of DNA methylation and Hippo signalling pathway during liver regeneration (PH) is still unclear. In this study, we investigated the regulatory mechanism of LATS1, a pivotal protein in the Hippo signalling pathway, on liver regeneration and explored the specific mechanism of DNA methylation regulating LATS1. To analyse the regulation of LATS1 by DNA methylation, following 2/3 partial hepatectomy (PH) in liver‐specific AAV‐8 shDNMT3B deleted mice (*DNMT3B*, KD) mice and sex‐matched AAV‐8 shControl (Control). We determined that DNMT3B regulates the protein expression of LATS1 by DNA methylation. miR‐29b‐3p significantly regulates the expression of DNMT3B and alters LATS1 expression to inactivate the Hippo signalling pathway, thereby reducing the expression of cell proliferation and cycle proteins and inhibiting liver regeneration. Our results indicated that the miR‐29b‐3p/DNMT3B regulatory axis influences LATS1 expression through DNA methylation, and thereby promotes liver regeneration.

## 1 Introduction

The liver is one of the few parenchymal organs with strong regenerative ability [1]. Hepatocytes account for approximately 65% of the total number of liver cells and are the ‘main force’ of liver regeneration (LR). Partial hepatectomy (PH) in rats or mice is the most commonly used model for studying liver regeneration [1, 2]. After partial liver lobectomy, the remaining liver lobes rapidly expand through a combination of liver cell hypertrophy and proliferation, compensating and restoring partial liver function [2]. Although the mechanism of liver regeneration has not been fully elucidated, it is clear that the Hippo signalling pathway is one of most important signalling pathways to regulate liver regeneration [3]. During liver regeneration, the cell proliferation and expression of apoptotic factors in the remaining liver cells are inevitably altered [4]. Hippo signalling pathway acts on the downstream effective factor Yes associated protein (YAP) through a series of kinase phosphorylation reactions, leading to its binding with cytoskeletal proteins and retention in the cytoplasm, thereby reducing its nuclear activity [5], through which the Hippo pathway controls organ size and volume by regulating cell proliferation and apoptosis [6, 7, 8, 9]. The regulatory role of Hippo pathway in liver regeneration has been confirmed in mouse models. Specific overexpression of YAP in the liver leads to liver enlargement, but once YAP overexpression is halted, liver size can return to normal [10, 11].

Large tumour suppressor kinase 1 (LATS1) plays an important role in the phosphorylation modification of YAP [12]. Konishi et al. found that LATS1 is inactivated during the initiation of liver regeneration, leading to non‐phosphorylation activation of YAP, upregulating the expression of connective tissue growth factor and apoptosis inhibitory protein, and promoting liver regeneration [13]. Loforese et al. found that during the termination stage of liver regeneration, LATS1 can induce phosphorylation of YAP, leading to a significant decrease in the expression level of cell proliferation factors regulated by YAP transcription [14]. The above suggests that the phosphorylation of YAP is crucial for regulating liver regeneration, while LATS1 plays an important role in the phosphorylation process of YAP.

DNA methylation is a common epigenetic modification, which is catalysed by methyltransferase to covalently bond to a methyl group at the cytosine (C) 5′ carbon position of CpG island dinucleotide in the genome, resulting in a conformational change in DNA and affecting its function [15, 16]. When DNA methylation occurs in the promoter region of a gene, it usually inhibits gene transcription [17]. During liver regeneration, the DNA methylation levels in the promoter regions of c‐myc, p53 and H‐RAS decrease, and protein expression significantly increases, promoting mitosis and proliferation of liver cells [18]. Transcriptomic analysis of PH in mice revealed that DNA hypomethylation caused by UHRF1 deficiency in liver cells can lead to sustained activation of genes that promote liver regeneration and enhance liver regeneration ability [19]. It can be seen that DNA methylation plays an important regulatory role in liver regeneration. In addition to classical protein phosphorylation modification, DNA methylation also plays a significant regulatory role in the protein expression of LATS1 [20]. A Previous study has confirmed that DNA methylation in the promoter region of LATS1 can inhibit its protein expression, leading to up regulation of cell proliferation and anti‐apoptotic factor expression, and promoting the proliferation and metastasis of breast cancer cells [21]. In liver cancer, DNA methylation in the LATS1 promoter region leads to dysfunction of the Hippo signalling pathway, inducing excessive production of monoacylglycerol lipase and promoting malignant proliferation of liver cancer cells [22]. However, the regulation of hepatocyte proliferation by DNA methylation and Hippo signalling pathway during LR and the molecular mechanism of these regulatory effects remains unclear.

In this study, we established liver‐specific DNMT3B‐deleted mice and found that DNMT3B regulates the expression level of LATS1 through DNA methylation after PH; furthermore, the results demonstrated that miR‐29b‐3p significantly regulates the expression of DNMT3B and alters LATS1 expression to inactivate the Hippo signalling pathway. In general, miR‐29b‐3p/DNMT3B regulatory axis influences LATS1 expression through DNA methylation, and regulates liver regeneration through the Hippo signalling pathway.

## 2 Materials and Methods

### 2.1 Mice and PH Model

The classical PH models was established as previously described [23]. The tissue and blood samples of each mouse were collected at indicated timepoints for histology and biochemical analysis, respectively. Nucleic acids and proteins extracted from liver tissues were further examination. Liver specifically DNMT3B knockdown mice were generated by AAV8‐shRNA DNMT3B. Male mice aged 8 ~ 10 weeks were subjected to hepatectomy. 5azaC was dissolved in sterile PBS and intraperitoneally injected into mice at 0.25 mg/kg once a day for 5 days starting 2 days before liver resection.

### 2.2 Western Blot

Extracting total protein from liver samples using RIPA lysis buffer (Beyotime). 25 μg of protein/well were electrophoresed by 10% sodium dodecyl sulphate polyacrylamide gel electrophoresis (SDS‐PAGE). PVDF membranes were blocked with 5% non‐fat powdered milk at room temperature for 1 h and incubated with primary antibody protein.

LATS1 (1:2000; Abcam), DNMT3B (1:3000; Abcam), p‐YAP, (1:1000; Abcam), YAP, (1:5000; Abcam), CDK1 (1:10000; Abcam), CDK4 (1:2000; Abcam), Cyclin A, (1:2000; Abcam), CyclinD1, (1:10000; Abcam), Cyclin E1, (1:1000; Abcam), and GAPDH antibody (1:5000, Abcam) at 4°C overnight. Chemiluminescence (ECL) (Thermo Fisher) was used to detect the expression of the target proteins.

### 2.3 Quantitative Real‐Time Polymerase Chain Reaction (RT‐qPCR)

Total RNAwas extracted by TRIzol reagent (TaKaRa) and reversely transcribed into cDNA using PrimeScript RT Reagent (TaKaRa). RT‐qPCR was performed using SYBR Premix Ex Taq II (TaKaRa). miR‐29b‐3p expression was determined by a TaqMan MicroRNA Assay kit (Applied Biosystems; Thermo Fisher Scientific Inc). U6 and GAPDH were used as internal references. The sequences used in this study are listed in Table S1.

### 2.4 Histology and Immunohistochemical Staining

Haematoxylin‐eosin (HE) staining and immunohistochemical staining to assess the pathological changes and the expression of LATS1 and Ki‐67 in mouse livers. The slices were incubated with LATS1(1:500; Abcam), and Ki‐67 (1:500; Abcam) overnight at 4°C, followed by biotinylated secondary antibody at 37°C for 1 h. Colour development was carried out with DAB (3,3‐diaminobenzidine). The slides were counterstained with 1% Mayer's haematoxylin. LATS1 and Ki‐67 immunostaining were scored and examined by two independent assessors.

### 2.5 Reintroduction of miR‐29b‐3p Agomir in Mice Liver

miR‐29b‐3p agomir (5 nmol/mouse) or an miRNA negative control (Ribo‐bio) was injected via tail vein into C57BL/6 mice at 1 day before 2/3 PH, and liver samples were collected at the designed experimental time points [23].

### 2.6 Bisulfite Sanger Sequencing (BSP)

For DNA methylaiton examination, 600 ng of genomic DNA extracted from liver samples was bisulfite converted using a MethylCode Bisulfite Conversion Kit (Applied Biosystems, USA). LATS1 promoter was amplified by PCR with Taq DNA Polymerase (Invitrogen, USA). The primer sequence was designed using Methyl Primer Express Software v1.0 (Applied Biosystems, USA). The PCR products were electrophoresed, purified using Spin‐X tubes, and then cloned into the pUC‐T vector (both from CWBiotech, Beijing, China). Ten single products were sequenced for each sample.

### 2.7 miRNA Target Prediction

Three prediction databases, including TargetScan (http://www.targetscan.org), Oncomir (http://www.oncomir.org/) and miRWalk (http://mirwalk.umm.uni‐heidelberg.de/) were used to predict miRNAs targeting DNMT3B.

### 2.8 Luciferase Reporter Assay

Wild‐type DNMT3B‐3′ UTR (wt) and mutant DNMT3B‐3′ UTR (mut) containing the putative binding site of miR‐29b‐3p were amplified by GenePharma and cloned into the firefly luciferase‐expressing pMIR‐REPORT vector (Obio Technology). Luciferase reporter vector and miR‐29b‐3p mimic, miR‐29b‐3p inhibitor and miR‐NC were transiently co‐transfected using Lipofectamine 3000. Luciferase assays were performed using the Luciferase Reporter Assay System (GloMax).

### 2.9 Statistical Analysis

All data are presented as the mean ± standard deviation (SD). SPSS 22.0 software and GraphPad Prism version were used for statistical analysis. Statistical differences were analysed by Student's *t*‐test, while the significance of differences between multiple groups was determined by one‐way analysis of variance, followed by the Newman–Keuls test, and repeated measures analysis of variance. *p* < 0.05 indicated statistically significant.

## 3 Results

### 3.1 LATS1 Regulates the Process of Liver Regeneration

**FIGURE 1:**
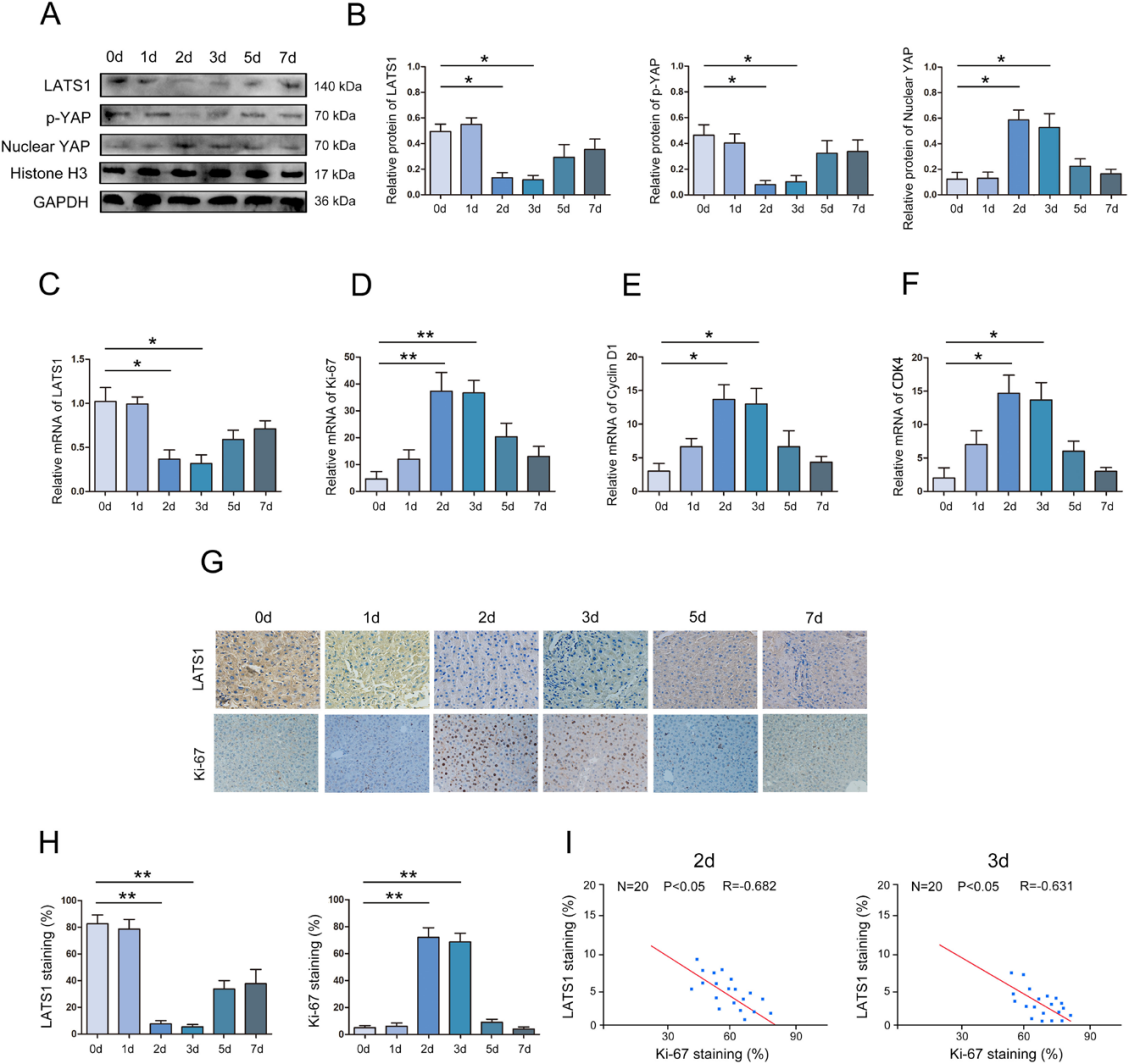
The expression of Hippo signalling pathway (LATS1, p‐YAP and Nuclear YAP) in liver regeneration. (A) Western blot was used to test the expression of LATS1, p‐YAP and Nuclear YAP in liver regeneration. (B) Statistical analysis of the expression of LATS1, p‐YAP and Nuclear YAP. (C) RT‐qPCR was used to analyse the expression of LATS1 in liver regeneration. (D) RT‐qPCR was used to analyse the expression of Ki‐67 in liver regeneration. (E) RT‐qPCR was used to analyse the expression of cyclin D1 in liver regeneration. (F) RT‐qPCR was used to analyse the expression of CKD4 in liver regeneration. (G) Immunohistochemistry was used to analyse the expression of LATS1 and Ki‐67 in liver regeneration, magnification X20. (H) Statistical analysis of the expression of LATS1 and Ki‐67 in liver regeneration. (I) Statistical correlation analysis of the expression relationship between LATS1 and Ki‐67. All data are represented as the mean ± SD, *p < 0.05, **p < 0.01.

We performed classic 2/3 PH in mice to determine the role of Hippo in liver regeneration. Western blot was used to analyse the expression of LATS1, p‐YAP and Nuclear YAP in the liver, and the results show that the expression of LATS1 and p‐YAP dynamic change (first decreased and then increased) during liver regeneration, while the expression of Nuclear YAP dynamic change (first increased and then decreased) (Figure 1A–C). In addition, cell proliferation‐related genes MKi‐67, Cyclin D1 and CDK4 show a reverse trend compared with LATS1 during liver regeneration (Figure 1D–F). IHC further confirmed the above results (Figure 1G,H). Moreover, statistical analysis showed that LATS1 was negatively correlated with Ki‐67 expression during the critical period of LR (Figure 1I).

### 3.2 DNA Methylation Regulates the Expression of LATS1 in Liver Regeneration

**FIGURE 2:**
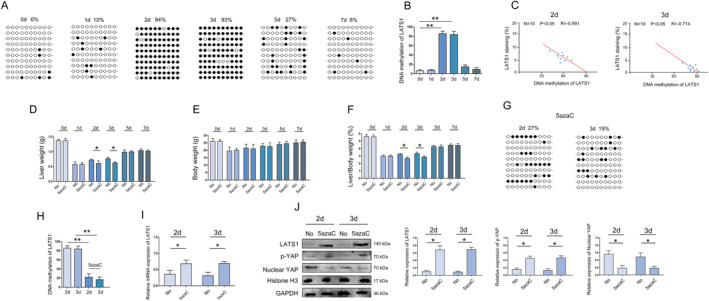
Detection and quantification of DNA methylation of LATS1 in regenerated liver. (A) Bisulfite sequencing analysis was performed on LATS1 promoter methylation in regenerated liver. Black dots, methylated; white dots, unmethylated. (B) Statistical analysis of DNA methylation level of LATS1 in liver regeneration. (C) Statistical correlation analysis of the relationship between LATS1 expression and DNA methylation. (D) Statistical analysis of the effect of demethylation treatment on liver weight during liver regeneration. (E) Statistical analysis of the effect of demethylation treatment on body weight during liver regeneration. (F) Statistical analysis of the effect of demethylation treatment on liver/body weight ratio during liver regeneration. (G) Bisulfite sequencing analysis was performed on LATS1 promoter methylation after demethylation treatment in regenerated liver. Black dots, methylated; white dots, unmethylated. (H) Statistical analysis of the effect of demethylation treatment on the expression of LATS1. (I) RT‐qPCR was used to analyse the expression of LATS1 after demethylation treatment in regenerated liver. (J) Western blot was used to analyse the expression of LATS1, p‐YAP and Nuclear YAP after demethylation treatment in regenerated liver. All data are represented as the mean ± SD, *p < 0.05, **p < 0.01.

The above results indicate that there is a significant difference in the expression of LATS1 during liver regeneration, and epigenetics can regulate gene expression without changing the nucleotide sequence of the gene [24]. Our previous study has also shown that DNA methylation plays a crucial role in the expression of LATS1 genes [20]. During liver regeneration, BSP detection results revealed the degree of methylation of LATS1 was gradually increased (Figure 2A,B). Moreover, statistical analysis showed that the methylation degree of LATS1 was negatively correlated with the expression of LATS1 (Figure 2C). A Previous study has reported that demethylating drugs can regulate DNA methylation levels of genes in liver regeneration [24]. Furthermore, we added demethylation drugs (5azaC) to treat mice and performed PH [25]. After demethylation, the remnant liver was sampled at 0, 1, 2, 3, 5 and 7 day time points for detection. In the early stage of liver regeneration (3 and 4 day), the liver/body weight ratio was lower (Figure 2D–F). The results showed that the methylation of LATS1 was significantly reduced and protein expression increased during liver regeneration (Figure 2G–I). The expression of p‐YAP was increased and Nuclear YAP was decreased during liver regeneration (Figure 2J).

### 3.3 DNMT3B Promotes Changes in LATS1 Methylation During Liver Regeneration

**FIGURE 3:**
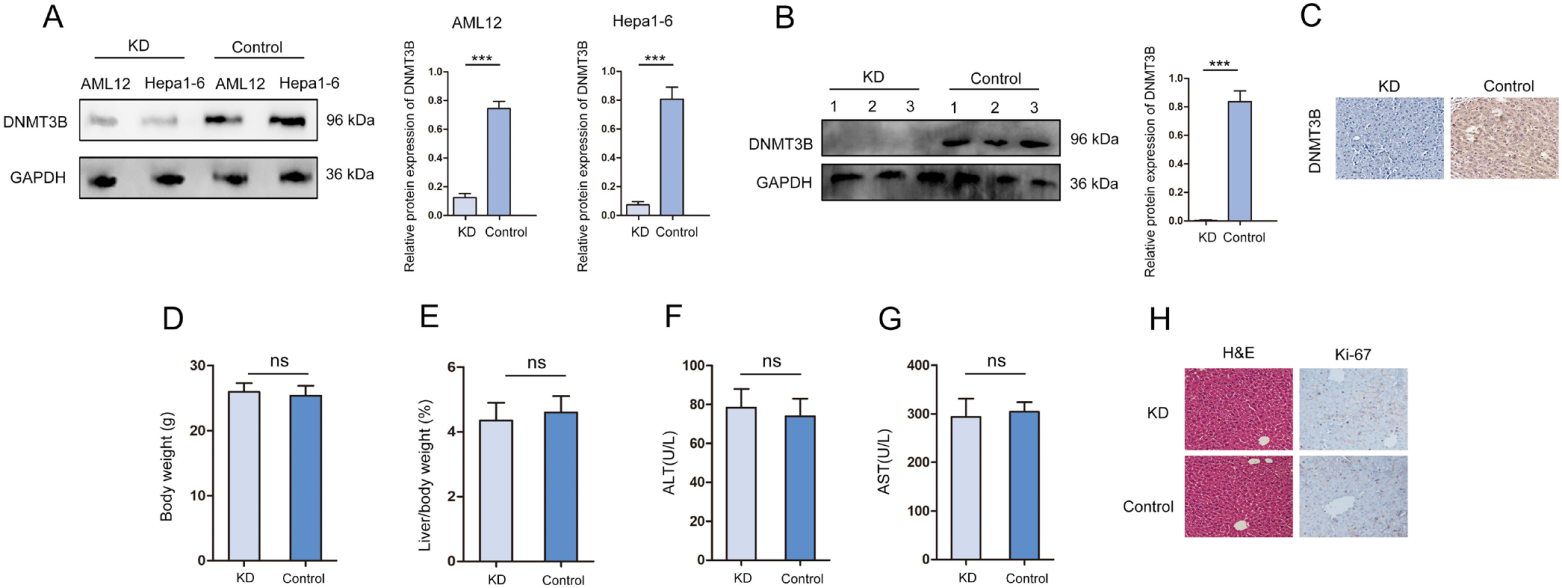
The construction of hepatocyte‐specific DNMT3B‐deficient mice. (A) The specific knockdown of DNMT3B in the liver and HCC cells. (B) Western blot was used to analyse the expression of DNMT3B in KD and Control mice. (C) Immunohistochemistry was used to analyse the expression of DNMT3B in KD and Control mice, magnification X20. (D) Body weight ratio results from KD and Control mice. (E) Liver/body weight ratio results from KD and Control mice. (F) The serological detection of ALT. (G) The serological detection of AST. (H) HE staining to test the liver condition of KD and Control mice, magnification X20. All data are represented as the mean ± SD, ***p < 0.001.

Methyltransferase is a key enzyme for DNA methylation, including DNMT1(maintain methylation), DNMT2 (methylated tRNA), DNMT3A (de novo methylation), and DNMT3B (de novo methylation) [25]. Our previous research found that the CpG island in the LATS1 promoter region can be catalysed by DNMT3B to undergo DNA methylation and inhibit the protein expression of LATS1 in HCC [20]. In addition, we further detected the expression of DNA methylation enzymes DNMT1, DNMT3A, and DNMT3B in liver regeneration, The results indicate that the expression trend of DNMT3B in liver regeneration is consistent with the change trend of LATS1 DNA methylation (Figure S1) To explore whether the DNA methylation of LATS1 regulated by DNMT3B during liver regeneration, we specifically deleted DNMT3B with AAV‐8 (KD). Shown in Figure 3A is the specific knockdown of DNMT3B in the liver and HCC cells. Western blot and immunohistochemical results show that DNMT3B was efficiently ablated in KD mouse livers (Figure 3B,C). Moreover, body weight, liver/weight ratio, ALT and AST results showed no significant difference in KD and Control mice (Figure 3D–G). The HE results showed no significant damage existed in KD mice (Figure 3H). These results indicated that L depletion of DNMT3B did not cause obvious liver injury.

**FIGURE 4:**
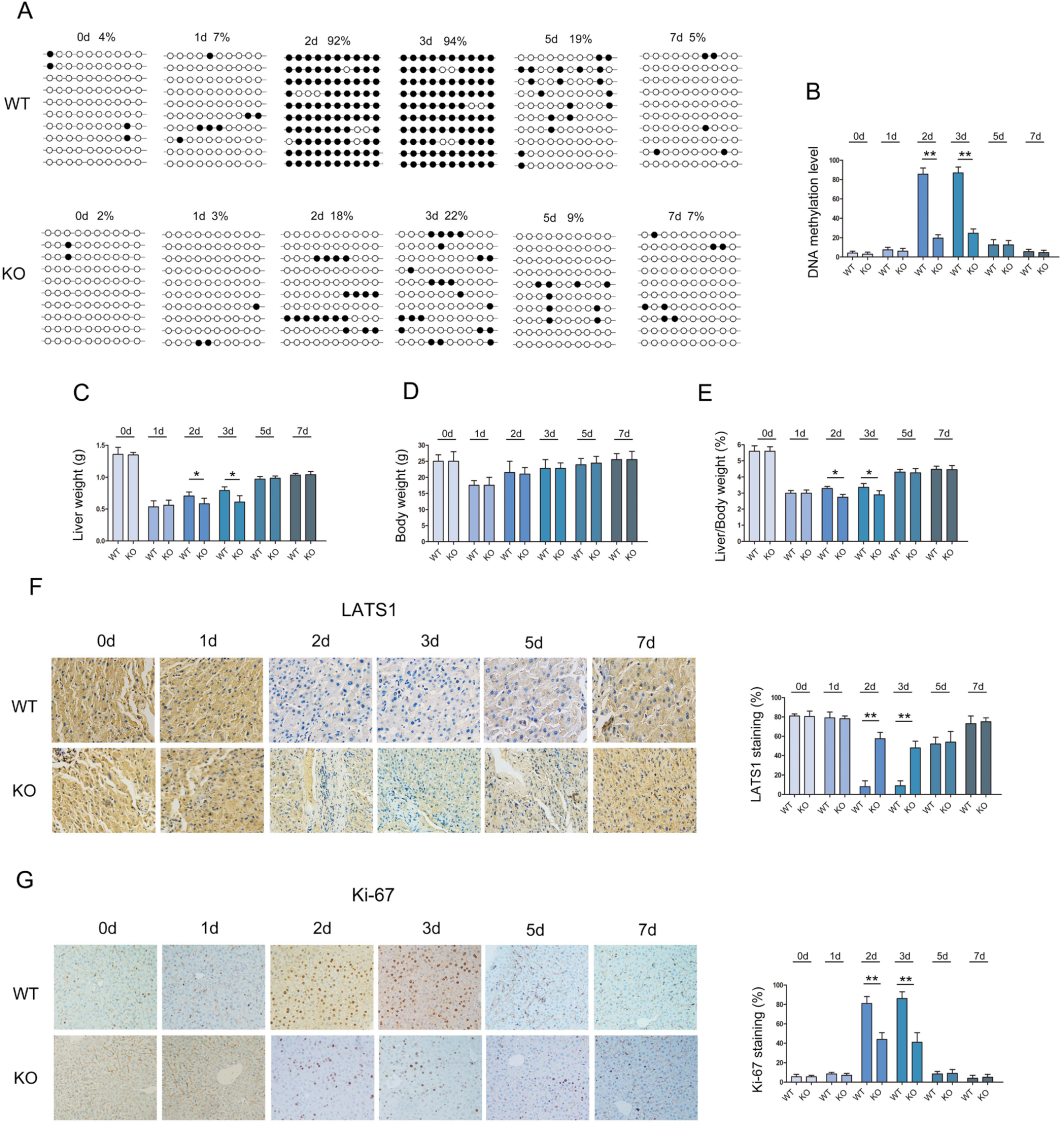
Detection of DNA methylation and protein of LATS1 in DNMT3B KD mice. (A) Statistical analysis of the effect of demethylation treatment on body weight during liver regeneration. (B) Statistical analysis of the effect of demethylation treatment on liver weight during liver regeneration. (C) Statistical analysis of the effect of demethylation treatment on liver/body weight ratio during liver regeneration. (D) Bisulfite sequencing analysis was performed on LATS1 promoter methylation in regenerated liver. Black dots, methylated; white dots, unmethylated. (E) Statistical analysis of DNA methylation of LATS1 in liver regeneration. (F) Immunohistochemistry was used to analyse the expression of LATS1 in KD and WT mice, magnification X20. (G) Immunohistochemistry was used to analyse the expression of Ki‐67 in KD and Control mice, magnification X20. *p < 0.05, **p < 0.01.

In the early stage of liver regeneration (2 and 3 days), the liver weight and liver/body weight ratio of KD mice were lower than that of Control mice (Figure 4A–C). In this period, DNA methylation of LATS1 in KD mice were lower than that of Control mice (Figure 4D,E). Additionally, the expression of LATS1 in KD mice was significantly higher than that in Control mice (Figure 4F). Moreover, the expression of Ki‐67 in KD mice was significantly lower than that in Control mice (Figure 4G).

**FIGURE 5:**
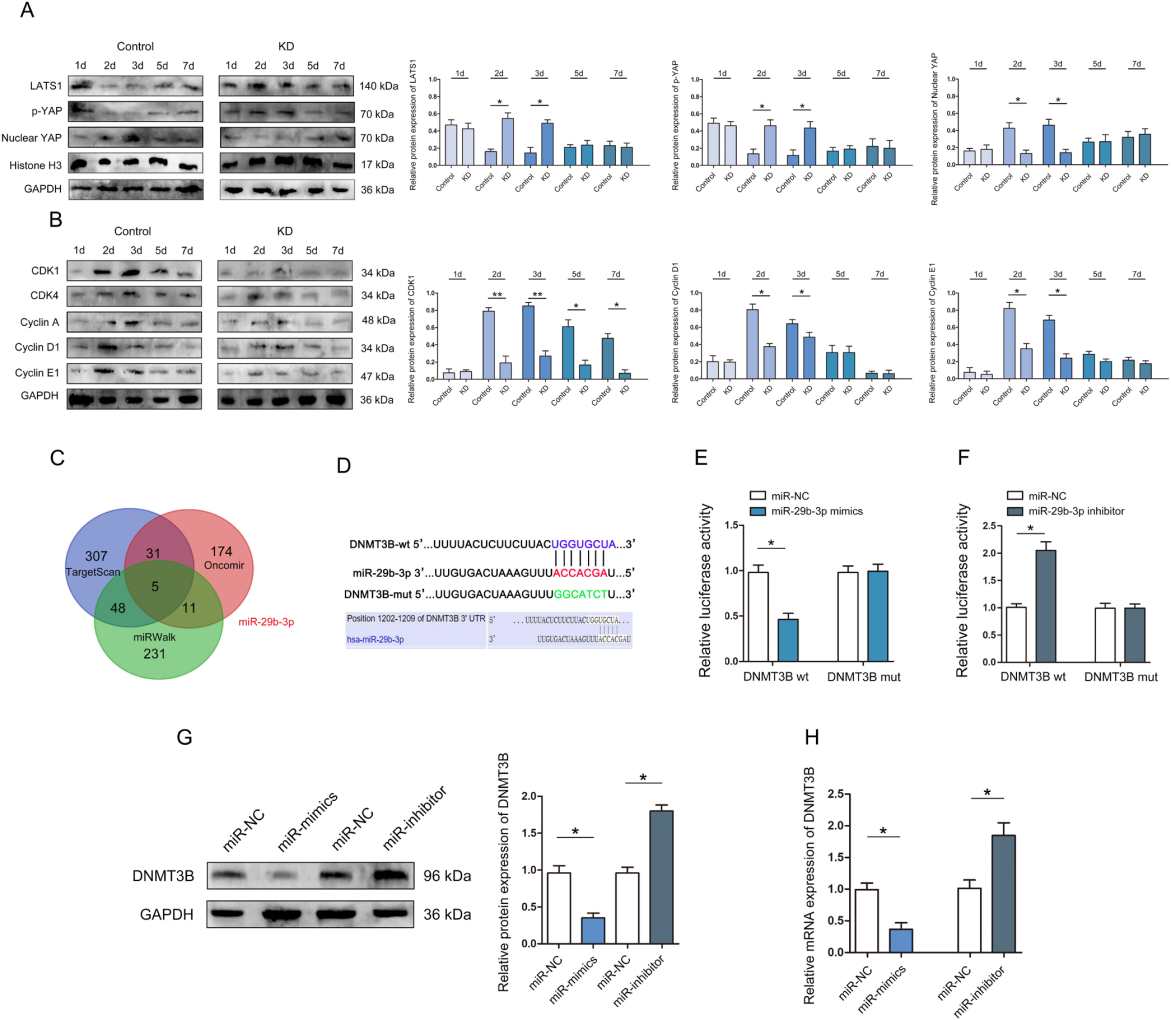
Detection and quantification of miR‐29b‐3p, cyclins, CDKs and key regulator proteins of Hippo signalling pathway in regenerated liver of KD and Control mice. (A) Western blot was used to analyse the expression of LATS1, p‐YAP and Nuclear YAP in liver regeneration. (B) CDK1, CDK4, cyclin A, cyclin D1 and cyclin E1 at indicated time points in liver regeneration. (C) Venn diagram displaying DNMT3B target miRNAs. (D) The binding sites and corresponding mutant sites of miR‐29b‐3p and DNMT3B 3′ UTR. (E) A dual luciferase activity assay was performed by cotransfection of luciferase reporter containing DNMT3B 3′ UTR or the mutant reporter with miR‐29b‐3p mimics. (F) A dual luciferase activity assay was performed by cotransfection of luciferase reporter containing DNMT3B 3′ UTR or the mutant reporter with miR‐29b‐3p inhibitor. (G) Protein expression of DNMT3B detected by western blotting. (H) The expression of DNMT3B detected by RT‐qPCR. *p < 0.05, **p < 0.01.

Western blot results show that LATS1 and p‐YAP were highly expressed in KD mouse livers, while Nuclear YAP was lower after 70% PH (Figure 5A). In addition, we found that CDK1, CDK4, cyclin A, cyclin D1 and cyclin E1 were all lower in KD compared to Control mice at early stage of liver regeneration (Figure 5B).

### 3.4 miR‐29b‐3p Regulates DNMT3B and Affects the DNA Methylation Level of LATS1 in Liver Regeneration

A large number of studies have shown that miRNA not only plays an important role in liver tumours, but also plays a broad regulatory role in liver regeneration [26]. TargetScan (http://www.targetscan.org), Oncomir (http://www.oncomir.org/) and miRWalk (http://mirwalk.umm.uni‐heidelberg.de/) were used to analyse upstream regulatory miRNAs of DNMT3B. The results show that miR‐29b‐3p were potential target gene of DNMT3B (Figure 5C). We detected the expression of miR‐29b‐3p during liver regeneration, and the results showed a significant correlation between miR‐29b‐3p and DNMT3B expression (Figure S1). Binding site analysis found that miR‐29b‐3p contained a potential binding site in DNMT3B (Figure 5D). Co‐transfection of miR‐29b‐3p mimics significantly decreased luciferase activity in cells transfected with wt DNMT3B 3′‐UTR (Figure 5E), and co‐transfection of miR‐29b‐3p inhibitor significantly increased luciferase activity in cells transfected with wt DNMT3B 3′‐UTR (Figure 5F). In addition, western blot and qRT–PCR assays revealed that miR‐29b‐3p overexpression significantly reduced DNMT3B protein and mRNA levels (Figure 5G,H). These results suggest that miR‐29b‐3p directly targets and downregulates DNMT3B.

**FIGURE 6:**
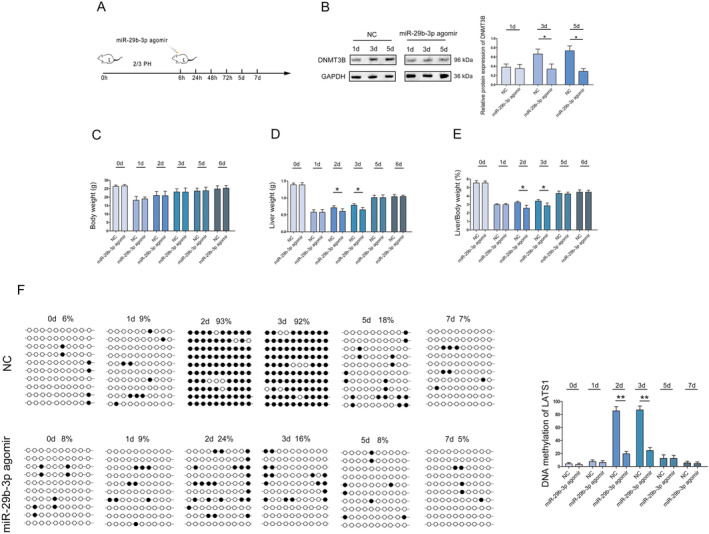
miR‐29b‐3p regulates DNMT3B and affects the DNA methylation of LATS1 in liver regeneration. (A) Schematic diagram for tail vein injection with miR‐29b‐5p agomir. (B) Western blot was used to analyse the expression of DNMT3B in liver regeneration. (C) Statistical analysis of the effect of demethylation treatment on body weight during liver regeneration. (D) Statistical analysis of the effect of demethylation treatment on liver weight during liver regeneration. (E) Statistical analysis of the effect of demethylation treatment on liver/ body weight ratio during liver regeneration. (F) Bisulfite sequencing analysis was performed on LATS1 promoter methylation in regenerated liver. Black dots, methylated; white dots, unmethylated. *p < 0.05, **p < 0.01.

Furthermore, we injected mice with the miR‐29b‐3p agomir via tail vein at 6 h after 2/3 PH, and the remnant liver was sampled at 0, 24, 48, 72 h, 5 and 7 day time points for detection (Figure 6A). Western blot results showed that DNMT3B expression in miR‐29b‐3p agomir mice were lower than that of NC mice (Figure 6B). In the early stage of liver regeneration (2 and 3 days), the liver weight and liver/body weight ratio of miR‐29b‐3p agomir mice were lower than that of NC mice (Figure 6C–E).

### 3.5 miR‐29b‐3p/DNM3B Regulatory Axis Regulates the Role of Hippo in Liver Regeneration Through LATS1

**FIGURE 7:**
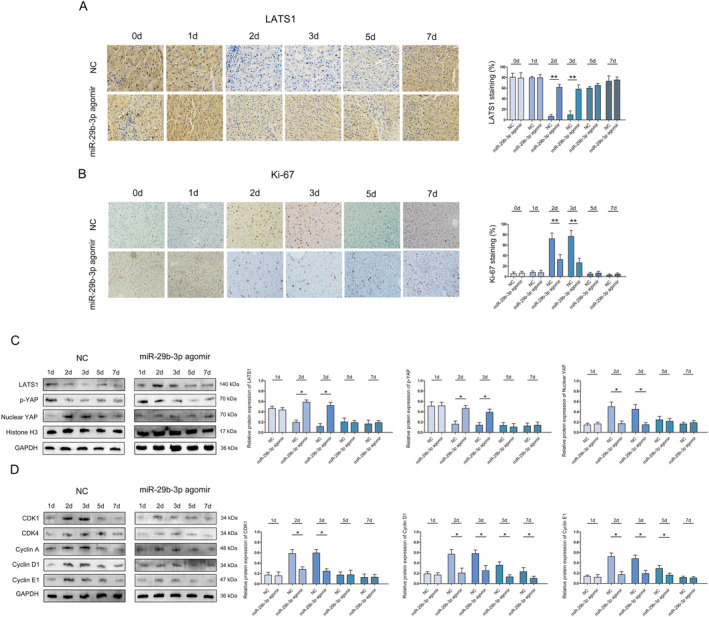
miR‐29b‐3p influences the role of Hippo signalling pathway in liver regeneration through LATS1. (A) Immunohistochemistry was used to analyse the expression of LATS1 in liver regeneration, magnification X20. (B) Immunohistochemistry was used to analyse the expression of Ki‐67 in liver regeneration, magnification X20. (C) Western blot was used to analyse the expression of LATS1, p‐YAP and Nuclear YAP in liver regeneration. (D) CDK1, CDK4, cyclin A, cyclin D1 and cyclin E1 at indicated time points in liver regeneration. *p < 0.05, **p < 0.01.

After increasing the expression of miR‐29b‐3p, the methylation of LATS1 was reduced (Figure 6F). In addition, at 2 and 3 days after PH, the expression of LATS1 in miR‐29b‐3p agomir mice was significantly higher than that in NC mice (Figure 7A). Moreover, the expression of Ki‐67 in miR‐29b‐3p agomir mice was significantly lower than that in NC mice (Figure 7B).

**FIGURE 8:**
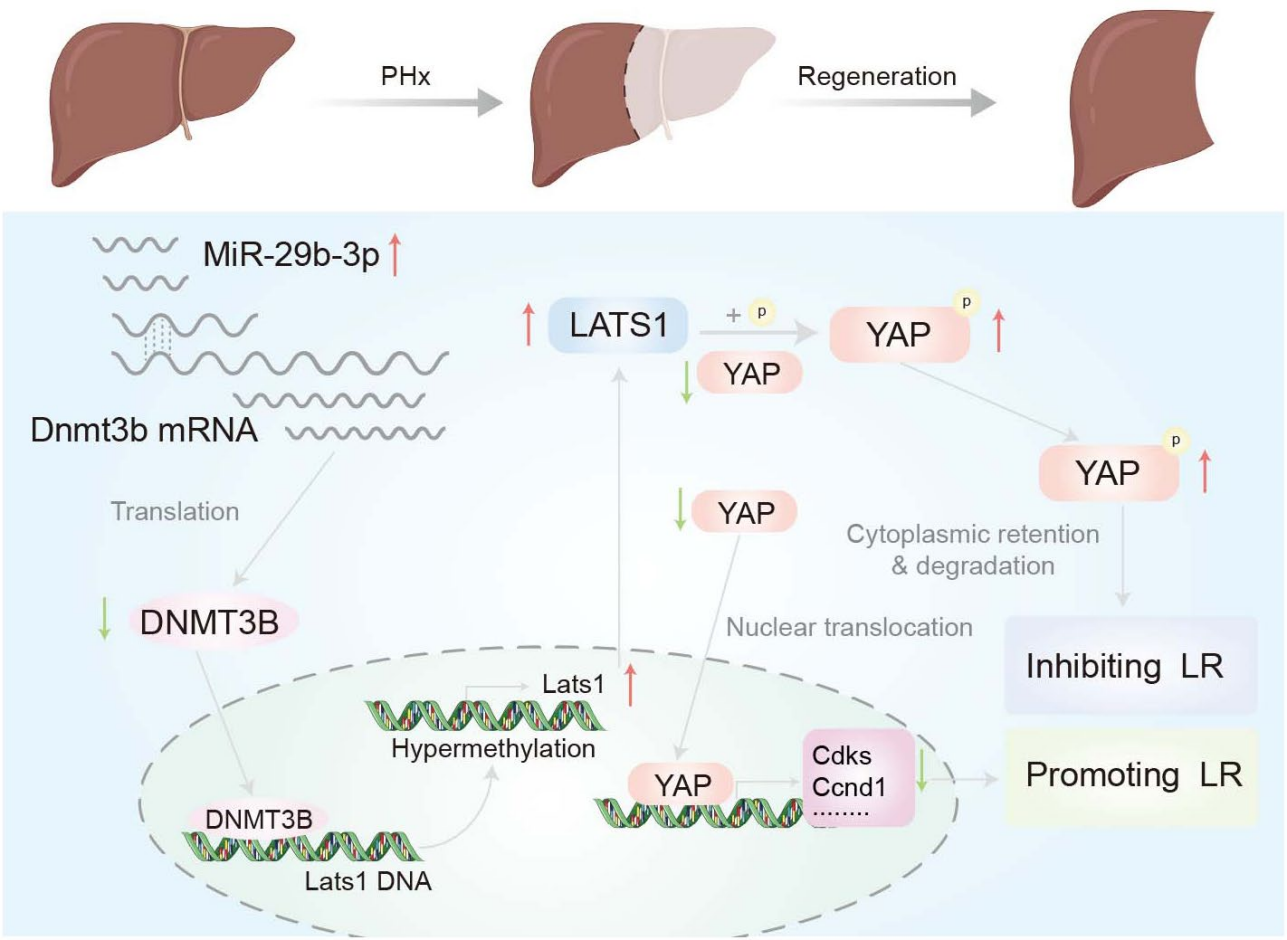
Schematic representation of miR‐29b‐3p reduces mice liver regeneration after PHx by down‐regulating the expression of DNMT3B and regulate the DNA methylation level of LATS1 through DNMT3B.

Western blot to detect the expression of LATS1, p‐YAP and Nuclear YAP in the process of liver regeneration at 1, 2, 3, 5 and 7 days. The results show that LATS1 and p‐YAP were highly expressed in miR‐29b‐3p agomir mouse livers, while YAP was lower after 70% PH (Figure 7C). In addition, we found that CDK1, CDK4, cyclin A, cyclin D1 and cyclin E1 were all lower in miR‐29b‐3p agomir mice compared to NC mice at early stage of liver regeneration (2 and 3 days) (Figure 7D). Overall, these results demonstrated that miR‐29b‐3p reduces mice liver regeneration after PHx by down‐regulating the expression of DNMT3B and regulate the DNA methylation of LATS1 (Figure 8).

## 4 Discussion

The Hippo signalling pathway is composed of a series of conserved kinases that regulate cell proliferation, apoptosis, stem cell self‐renewal and participate in multiple biological functions such as tissue development, tissue homeostasis maintenance and regeneration repair in various organisms [27, 28]. When Hippo pathway is activated, the activity of YAP/TAZ is inhibited through LATS1/2 mediated phosphorylation [29]. When Hippo pathway is inactivated, the dephosphorylated YAP/TAZ translocation enters the nucleus and binds to the transcription factor TEAD1‐4 to induce gene expression [30]. Hippo signalling pathway plays an important role in liver regeneration: on the one hand, it activates cell proliferation and the expression of anti‐apoptotic factors in the initiation and maintenance of liver regeneration to promote liver regeneration; On the other hand, reducing the expression of cell proliferation and anti‐apoptotic factors during the termination stage of liver regeneration inhibits liver regeneration [31]. Our study found that LATS1, a key protein of Hippo signalling pathway, CpG island in LATS1 promoter region can undergo DNA methylation changes (the degree of DNA methylation first increases and then gradually decreases) during liver regeneration, and significantly regulates its protein expression. Dynamic changes in cell proliferation and anti‐apoptotic factor expression regulated by YAP transcription (initially increasing and then gradually decreasing).

Methyltransferase is a key enzyme involved in DNA methylation of genes [32]. There are currently four known human methyltransferases: DNMT1, DNMT2, DNMT3A, and DNMT3B, which play important roles in de novo methylation and retention methylation processes, respectively [33]. Research has shown that DNMT3A is consistently expressed at low levels at different stages of the cell cycle, while DNMT2 is mainly involved in tRNA methylation modification rather than DNA methylation. However, DNMT1 and DNMT3B are induced to be highly expressed in the S phase, providing methylation modification guarantees for DNA replication, transcription, and protein synthesis [34]. Research has shown that DNMT3A is consistently expressed at low levels at different stages of the cell cycle, while DNMT1 and DNMT3B are induced to be highly expressed in the S phase, providing methylation modifications for DNA replication, transcription, and protein synthesis after liver injury, the number of hepatocytes decreases sharply, and various feedback signals stimulate the proliferation of hepatocytes in the G0 stage, and residual hepatocytes change from basically non‐growth state to rapid growth state through cell proliferation, so as to compensate for the loss and damage of liver tissue and restore the physiological function of the liver [35]. Research has shown that DNMT3B is highly expressed in the S phase of cell proliferation, providing methylation modification guarantees for DNA replication, transcription, and protein synthesis [36]. Liver regeneration is a process of liver cell proliferation and differentiation, where gene sequences undergo de novo methylation to produce new DNA methylation modifications, and DNMT3B is the key methyltransferase for de novo methylation [37, 38]. In our existing studies, we found that the expression of DNMT3B changed dynamically during liver regeneration (increased and then decreased gradually), and DNMT3B could significantly regulate the DNA methylation and protein expression of LATS1.

microRNAs (miRNAs) exert their functions by regulating the expression of specific target genes and are related to various pathophysiological activities of liver [39]. Some miRNAs have also been shown to be involved in the regulation of liver regeneration [40]. During the initiation stage of liver regeneration, the expression of miR‐21 is significantly upregulated at 1, 6, 12, 24 and 48 h, and reaches its peak at 12 and 24 h [41]. At the same time, it can promote the translation of cyclin D1 and initiate liver cell proliferation [41]. The expression of miR‐127 is significantly reduced during the initiation stage of liver regeneration [42]. Mechanism studies have shown that miR‐127 can promote the expression of Bcl‐6 and promote liver cell proliferation [42]. During the phase of liver regeneration and proliferation, miR‐221 regulates liver cell proliferation by influencing aromatic hydrocarbon nuclear transferors and subsequently regulating the expression of p27.^43^ During the termination stage of liver regeneration, miR‐23b inhibits the expression of TGF‐b1 through Smad3, and promotes the expression of TGF‐β, activing A and inhibits the proliferation of liver cells.^44^ Our results are also consistent with previous studies. Furthermore, we found that miR‐29b‐3p regulates the DNA methylation modification of LATS1 by regulating DNMT3B, inhibits the expression of LATS1, and dynamically regulates the process of liver regeneration.

In summary, this study provides evidence that miR‐29b‐3p reduces mice liver regeneration after PHx by down‐regulating the expression of DNMT3B and regulate the DNA methylation of LATS1 through DNMT3B, consequently leads to affecting the liver regeneration process by regulating the function of Hippo signalling pathway.

## Author Contributions

**Yinwen Zhou:** conceptualization (equal), data curation (equal), methodology (equal), writing – original draft (equal), writing – review and editing (equal). **Hao Wu:** conceptualization (equal), formal analysis (equal), funding acquisition (equal), methodology (equal), writing – original draft (equal), writing – review and editing (equal). **Qiu Wang:** conceptualization (equal), investigation (equal), project administration (equal), resources (equal), software (equal), supervision (equal), validation (equal). **Bo Ma:** formal analysis (equal), funding acquisition (equal), investigation (equal), methodology (equal), project administration (equal), resources (equal), software (equal), supervision (equal), validation (equal), visualization (equal). **Jiulong Sun:** funding acquisition (equal), project administration (equal), resources (equal), software (equal), validation (equal), visualization (equal). **Guoliang Wang:** conceptualization (equal), funding acquisition (equal), writing – original draft (equal), writing – review and editing (equal).

## Ethics Statement

The Animal Care and Use Committee of Guizhou Provincial People's Hospital granted approval for all experiments involving animals.

## Consent

All authors consent to publication.

## Conflicts of Interest

The authors declare no conflicts of interest.

## Supporting information


**Figure S1.** Western blot was used to analyse the expression of DNMT1, DNMT3A and DNMT3B in liver regeneration. **p < 0.01.

**Figure S2.** RT‐qPCR was used to analyse the expression of miR‐29b‐3p in liver regeneration.

**Table S1.** The sequences of RT‐qPCR.

## Data Availability

The data that support the findings of this study are available on request from the corresponding author. The data are not publicly available due to privacy or ethical restrictions.

## References

[1] G. K. Michalopoulos and B. Bhushan , “Liver Regeneration: Biological and Pathological Mechanisms and Implications,” Nature Reviews. Gastroenterology & Hepatology 18, no. 1 (2021): 40–55. 32764740 10.1038/s41575-020-0342-4

[2] S. A. Mao , J. M. Glorioso , and S. L. Nyberg , “Liver Regeneration,” Translational Research 163, no. 4 (2014): 352–362. 24495569 10.1016/j.trsl.2014.01.005 PMC3976740

[3] J. O. Russell and F. D. Camargo , “Hippo Signalling in the Liver: Role in Development, Regeneration and Disease,” Nature Reviews. Gastroenterology & Hepatology 19, no. 5 (2022): 297–312. 35064256 10.1038/s41575-021-00571-w PMC9199961

[4] J. L. Duan , B. Ruan , P. Song , et al., “Shear Stress‐Induced Cellular Senescence Blunts Liver Regeneration Through Notch‐Sirtuin 1‐P21/P16 Axis,” Hepatology 75, no. 3 (2022): 584–599. 34687050 10.1002/hep.32209

[5] S. Ma , Z. Meng , R. Chen , and K. L. Guan , “The Hippo Pathway: Biology and Pathophysiology,” Annual Review of Biochemistry 88 (2019): 577–604. 10.1146/annurev-biochem-013118-111829 30566373

[6] Z. Meng , T. Moroishi , and K. L. Guan , “Mechanisms of Hippo Pathway Regulation,” Genes & Development 30, no. 1 (2016): 1–17. 26728553 10.1101/gad.274027.115 PMC4701972

[7] Z. Wu and K. L. Guan , “Hippo Signaling in Embryogenesis and Development,” Trends in Biochemical Sciences 46, no. 1 (2021): 51–63. 32928629 10.1016/j.tibs.2020.08.008 PMC7749079

[8] I. M. Moya and G. Halder , “Hippo‐YAP/TAZ Signalling in Organ Regeneration and Regenerative Medicine,” Nature Reviews. Molecular Cell Biology 20, no. 4 (2019): 211–226. 30546055 10.1038/s41580-018-0086-y

[9] S. Piccolo , S. Dupont , and M. Cordenonsi , “The Biology of YAP/TAZ: Hippo Signaling and Beyond,” Physiological Reviews 94, no. 4 (2014): 1287–1312. 25287865 10.1152/physrev.00005.2014

[10] F. D. Camargo , “YAP1 Increases Organ Size and Expands Undifferentiated Progenitor Cells,” Current Biology 17 (2007): 2054–2060. 17980593 10.1016/j.cub.2007.10.039

[11] J. Dong , “Elucidation of a Universal Size‐Control Mechanism in Drosophila and Mammals,” Cell 130 (2007): 1120–1133. 17889654 10.1016/j.cell.2007.07.019 PMC2666353

[12] A. W. Hong , Z. Meng , and K. L. Guan , “The Hippo Pathway in Intestinal Regeneration and Disease,” Nature Reviews. Gastroenterology & Hepatology 13, no. 6 (2016): 324–337. 27147489 10.1038/nrgastro.2016.59 PMC5642988

[13] T. Konishi , R. M. Schuster , and A. B. Lentsch , “Proliferation of Hepatic Stellate Cells, Mediated by YAP and TAZ, Contributes to Liver Repair and Regeneration After Liver Ischemia‐Reperfusion Injury,” American Journal of Physiology. Gastrointestinal and Liver Physiology 314, no. 4 (2018): G471–G482. 29351389 10.1152/ajpgi.00153.2017 PMC5966748

[14] G. Loforese , T. Malinka , A. Keogh , et al., “Impaired Liver Regeneration in Aged Mice Can Be Rescued by Silencing Hippo Core Kinases MST1 and MST2,” EMBO Molecular Medicine 9, no. 1 (2017): 46–60. 27940445 10.15252/emmm.201506089 PMC5210079

[15] A. L. Mattei , N. Bailly , and A. Meissner , “DNA Methylation: A Historical Perspective,” Trends in Genetics 38, no. 7 (2022): 676–707. 35504755 10.1016/j.tig.2022.03.010

[16] L. D. Moore , T. Le , and G. Fan , “DNA Methylation and Its Basic Function,” Neuropsychopharmacology 38, no. 1 (2013): 23–38. 22781841 10.1038/npp.2012.112 PMC3521964

[17] H. Meng , Y. Cao , J. Qin , et al., “DNA Methylation, Its Mediators and Genome Integrity,” International Journal of Biological Sciences 11, no. 5 (2015): 604–617. 25892967 10.7150/ijbs.11218 PMC4400391

[18] M. Arechederra , C. Berasain , M. A. Avila , and M. G. Fernández‐Barrena , “Chromatin Dynamics During Liver Regeneration,” Seminars in Cell & Developmental Biology 97 (2020): 38–46. 30940574 10.1016/j.semcdb.2019.03.004

[19] S. Wang , C. Zhang , D. Hasson , et al., “Epigenetic Compensation Promotes Liver Regeneration,” Developmental Cell 50, no. 1 (2019): 43–56.e6. 31231040 10.1016/j.devcel.2019.05.034 PMC6615735

[20] H. Wu , W. Zhang , Z. Wu , et al., “miR‐29c‐3p Regulates DNMT3B and LATS1 Methylation to Inhibit Tumor Progression in Hepatocellular Carcinoma,” Cell Death & Disease 10, no. 2 (2019): 48. 30718452 10.1038/s41419-018-1281-7 PMC6362005

[21] Y. Takahashi , Y. Miyoshi , C. Takahata , et al., “Down‐Regulation of LATS1 and LATS2 mRNA Expression by Promoter Hypermethylation and Its Association With Biologically Aggressive Phenotype in Human Breast Cancers,” Clinical Cancer Research 11, no. 4 (2005): 1380–1385. 15746036 10.1158/1078-0432.CCR-04-1773

[22] J. Zhang , Z. Liu , Z. Lian , et al., “Monoacylglycerol Lipase: A Novel Potential Therapeutic Target and Prognostic Indicator for Hepatocellular Carcinoma,” Scientific Reports 6 (2016): 35784. 27767105 10.1038/srep35784 PMC5073346

[23] J. Gong , T. Mou , H. Wu , and Z. Wu , “Brg1 Regulates Murine Liver Regeneration by Targeting miR‐187‐5p Dependent on Hippo Signalling Pathway,” Journal of Cellular and Molecular Medicine 24, no. 19 (2020): 11592–11602. 32845093 10.1111/jcmm.15776 PMC7576256

[24] C. Ito , R. Haraguchi , K. Ogawa , et al., “Demethylation in Promoter Region of Severely Damaged Hepatocytes Enhances Chemokine Receptor CXCR4 Gene Expression,” Histochemistry and Cell Biology 160, no. 5 (2023): 407–418. 37532885 10.1007/s00418-023-02229-x

[25] C. Hu , X. Liu , Y. Zeng , J. Liu , and F. Wu , “DNA Methyltransferase Inhibitors Combination Therapy for the Treatment of Solid Tumor: Mechanism and Clinical Application,” Clinical Epigenetics 13, no. 1 (2021): 166. 34452630 10.1186/s13148-021-01154-x PMC8394595

[26] M. L. Finch , J. U. Marquardt , G. C. Yeoh , and B. A. Callus , “Regulation of microRNAs and Their Role in Liver Development, Regeneration and Disease,” International Journal of Biochemistry & Cell Biology 54 (2014): 288–303. 24731940 10.1016/j.biocel.2014.04.002

[27] A. Ardestani , B. Lupse , and K. Maedler , “Hippo Signaling: Key Emerging Pathway in Cellular and Whole‐Body Metabolism,” Trends in Endocrinology and Metabolism 29, no. 7 (2018): 492–509. 29739703 10.1016/j.tem.2018.04.006

[28] J. Huang , S. Wu , J. Barrera , K. Matthews , and D. Pan , “The Hippo Signaling Pathway Coordinately Regulates Cell Proliferation and Apoptosis by Inactivating Yorkie, the Drosophila Homolog of YAP,” Cell 122, no. 3 (2005): 421–434. 16096061 10.1016/j.cell.2005.06.007

[29] I. M. Moya and G. Halder , “The Hippo Pathway in Cellular Reprogramming and Regeneration of Different Organs,” Current Opinion in Cell Biology 43 (2016): 62–68. 27592171 10.1016/j.ceb.2016.08.004

[30] A. Valizadeh , M. Majidinia , H. Samadi‐Kafil , M. Yousefi , and B. Yousefi , “The Roles of Signaling Pathways in Liver Repair and Regeneration,” Journal of Cellular Physiology 234, no. 9 (2019): 14966–14974. 30770551 10.1002/jcp.28336

[31] C. Yan , H. Yang , P. Su , et al., “OTUB1 Suppresses Hippo Signaling via Modulating YAP Protein in Gastric Cancer,” Oncogene 41, no. 48 (2022): 5186–5198. 36271031 10.1038/s41388-022-02507-3 PMC9700521

[32] D. Mangnall , N. C. Bird , and A. W. Majeed , “The Molecular Physiology of Liver Regeneration Following Partial Hepatectomy,” Liver International 23, no. 2 (2003): 124–138. 12654135 10.1034/j.1600-0676.2003.00812.x

[33] F. Lyko , “The DNA Methyltransferase Family: A Versatile Toolkit for Epigenetic Regulation,” Nature Reviews. Genetics 19, no. 2 (2018): 81–92. 10.1038/nrg.2017.80 29033456

[34] Z. Chen and Y. Zhang , “Role of Mammalian DNA Methyltransferases in Development,” Annual Review of Biochemistry 89 (2020): 135–158. 10.1146/annurev-biochem-103019-102815 31815535

[35] M. Gagliardi , M. Strazzullo , and M. R. Matarazzo , “DNMT3B Functions: Novel Insights From Human Disease,” Frontiers in Cell and Development Biology 6 (2018): 140. 10.3389/fcell.2018.00140 PMC6204409 30406101

[36] M. Okano , D. W. Bell , D. A. Haber , and E. Li , “DNA Methyltransferases Dnmt3a and Dnmt3b Are Essential for De Novo Methylation and Mammalian Development,” Cell 99, no. 3 (1999): 247–257. 10555141 10.1016/s0092-8674(00)81656-6

[37] F. Schueller , S. Roy , M. Vucur , C. Trautwein , T. Luedde , and C. Roderburg , “The Role of miRNAs in the Pathophysiology of Liver Diseases and Toxicity,” International Journal of Molecular Sciences 19, no. 1 (2018): 261. 29337905 10.3390/ijms19010261 PMC5796207

[38] X. Chen , Y. Zhao , F. Wang , Y. Bei , J. Xiao , and C. Yang , “MicroRNAs in Liver Regeneration,” Cellular Physiology and Biochemistry 37, no. 2 (2015): 615–628. 26344368 10.1159/000430381

[39] E. uskeviciute , R. P. Dippold , A. N. Antony , A. Swarup , R. Vadigepalli , and J. B. Hoek , “Inhibition of miR‐21 Rescues Liver Regeneration After Partial Hepatectomy in Ethanol‐Fed Rats,” American Journal of Physiology. Gastrointestinal and Liver Physiology 311, no. 5 (2016): G794–G806. 27634014 10.1152/ajpgi.00292.2016 PMC5130549

[40] C. Pan , H. Chen , L. Wang , et al., “Down‐Regulation of MiR‐127 Facilitates Hepatocyte Proliferation During Rat Liver Regeneration,” PLoS One 7, no. 6 (2012): e39151. 22720056 10.1371/journal.pone.0039151 PMC3376093

[41] Q. Yuan , K. Loya , B. Rani , et al., “MicroRNA‐221 Overexpression Accelerates Hepatocyte Proliferation During Liver Regeneration,” Hepatology 57, no. 1 (2013): 299–310. 22821679 10.1002/hep.25984

[42] B. Yuan , R. Dong , D. Shi , et al., “Down‐Regulation of miR‐23b May Contribute to Activation of the TGF‐β1/Smad3 Signalling Pathway During the Termination Stage of Liver Regeneration,” FEBS Letters 585, no. 6 (2011): 927–934. 21354414 10.1016/j.febslet.2011.02.031

