# Supplementary figures and images for: DNA Methylation Regulatory Axis miR‐29b‐3p/DNMT3B Regulates Liver Regeneration Process by Altering LATS1
Source: J Cell Mol Med. 2025 Feb 12;29(3):e70405. doi: 10.1111/jcmm.70405 (PMC11816157; doi:10.1111/jcmm.70405)

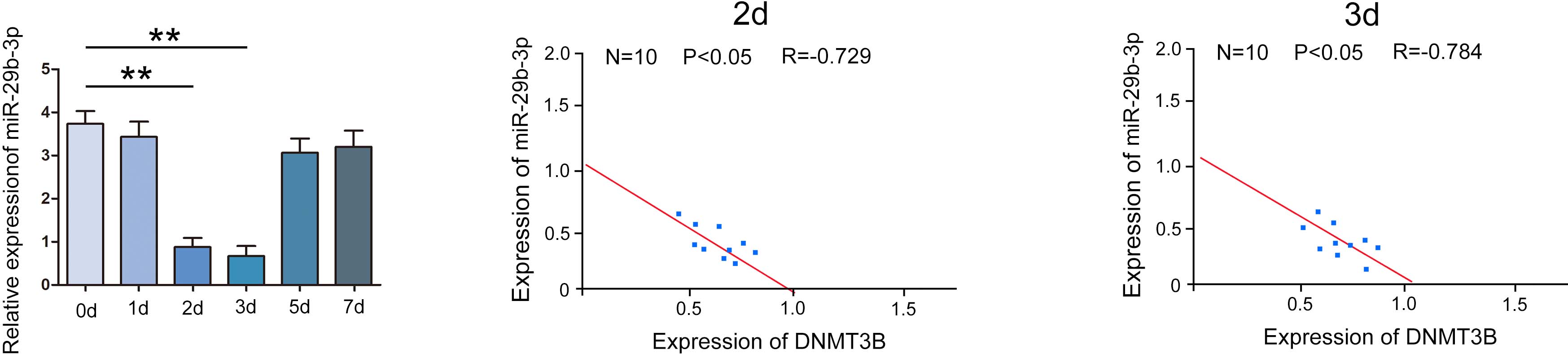

Supplement: Supplementary file 1 — Figure S1. Western blot was used to analyse the expression of DNMT1, DNMT3A and DNMT3B in liver regeneration. **p < 0.01. [file JCMM-29-e70405-s001.jpg]

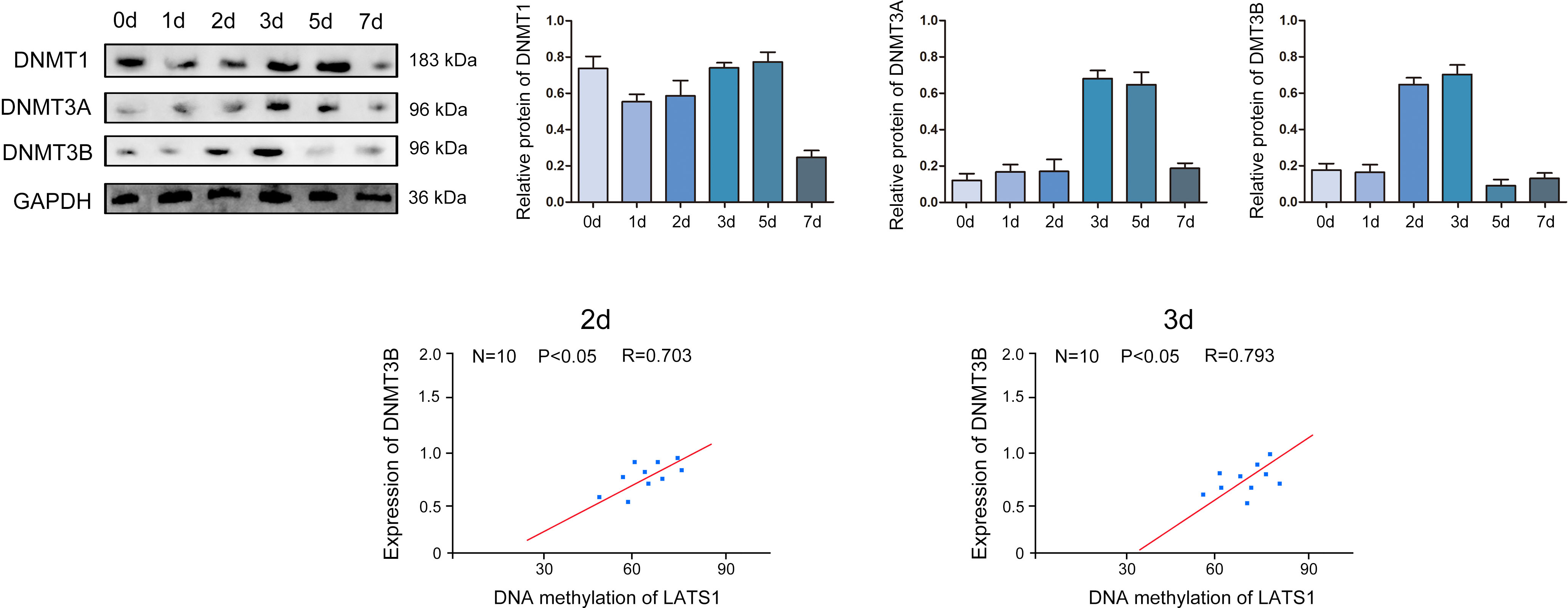

Supplement: Supplementary file 2 — Figure S2. RT‐qPCR was used to analyse the expression of miR‐29b‐3p in liver regeneration. [file JCMM-29-e70405-s003.jpg]
