# Supplementary material for: DNA Methylation Regulatory Axis miR‐29b‐3p/DNMT3B Regulates Liver Regeneration Process by Altering LATS1
Source: J Cell Mol Med. 2025 Feb 12;29(3):e70405. doi: 10.1111/jcmm.70405 (PMC11816157; doi:10.1111/jcmm.70405)
Supplement: Supplementary file 3 — Table S1. The sequences of RT‐qPCR. [file JCMM-29-e70405-s002.docx]

LATS1:

Forward Primer 5' -TGG TGA CTC TGG GGA TAA AGA A- 3'

Reverse Primer 5' –GGG AGT AAC TCT GAA TCC GAG AC- 3'

Ki-67

Forward Primer 5' –ATC ATT GAC CGC TCC TTT AGG T- 3'

Reverse Primer 5' –GCT CGC CTT GAT GGT TCC T- 3'

Cyclin D1

Forward Primer 5' –GCG TAC CCT GAC ACC AAT CTC- 3'

Reverse Primer 5' –CTC CTC TTC GCA CTT CTG CTC- 3'

CDK4

Forward Primer 5' –ATG GCT GCC ACT CGA TAT GAA- 3'

Reverse Primer 5' –TCC TCC ATT AGG AAC TCT CAC AC- 3'
